# A method for estimating the power of moments

**DOI:** 10.1186/s13660-018-1645-7

**Published:** 2018-03-06

**Authors:** Shuhua Chang, Deli Li, Yongcheng Qi, Andrew Rosalsky

**Affiliations:** 1 Coordinated Innovation Center for Computable Modeling in Management Science, Tianjin University of Finance and Economics, Tianjin, China; 2 Department of Mathematical Sciences, Lakehead University, Thunder Bay, Canada; 3 Department of Mathematics and Statistics, University of Minnesota Duluth, Duluth, USA; 4 Department of Statistics, University of Florida, Gainesville, USA

**Keywords:** 62F10, 60F15, 62F12, Asymptotic theorems, Consistent estimator, Point estimator, Power of moments

## Abstract

Let *X* be an observable random variable with unknown distribution function $F(x) = \mathbb{P}(X \leq x)$, $- \infty< x < \infty$, and let

$$\theta= \sup\bigl\{ r \geq0: \mathbb{E} \vert X \vert ^{r} < \infty\bigr\} . $$

We call *θ* the power of moments of the random variable *X*. Let $X_{1}, X_{2}, \ldots, X_{n}$ be a random sample of size *n* drawn from $F(\cdot)$. In this paper we propose the following simple point estimator of *θ* and investigate its asymptotic properties:

$$\hat{\theta}_{n} = \frac{\log n}{\log\max_{1 \leq k \leq n} \vert X_{k} \vert }, $$

where $\log x = \ln(e \vee x)$, $- \infty< x < \infty$. In particular, we show that

$$\hat{\theta}_{n} \rightarrow_{\mathbb{P}} \theta\quad\mbox{if and only if}\quad\lim_{x \rightarrow\infty} x^{r} \mathbb{P}\bigl( \vert X \vert > x\bigr) = \infty\quad\forall r > \theta. $$

This means that, under very reasonable conditions on $F(\cdot)$, $\hat {\theta}_{n}$ is actually a consistent estimator of *θ*.

## Motivation

The motivation of the current work arises from the following problem concerning parameter estimation. Let *X* be an observable random variable with unknown distribution function $F(x) = \mathbb{P}(X \leq x)$, $- \infty< x < \infty$, and let

$$\theta= \sup\bigl\{ r \geq0: \mathbb{E} \vert X \vert ^{r} < \infty\bigr\} . $$

We call *θ* the *power of moments* of the random variable *X*. Clearly *θ* is a parameter of the distribution of the random variable *X*. Now let $X_{1}, X_{2}, \ldots, X_{n}$ be a random sample of size *n* drawn from the random variable *X*; i.e., $X_{1}, X_{2}, \ldots, X_{n}$ are independent and identically distributed (i.i.d.) random variables whose common distribution function is $F(\cdot)$. It is natural to pose the following question: Can we estimate the parameter *θ* based on the random sample $X_{1}$, …, $X_{n}$?

This is a serious and important problem. For example, if $\theta> 2$ and if the distribution of *X* is nondegenerate, then it is clear that $0 < \operatorname{Var} X < \infty $ and so by the classical Lévy central limit theorem, the distribution of

$$\frac{S_{n} - n \mu}{\sqrt{n}} $$

is approximately normal (for all sufficiently large *n*) with mean 0 and variance $\sigma^{2} = \operatorname{Var}X = \mathbb{E}(X - \mu)^{2}$ where $\mu = \mathbb{E}X$. Thus the problem that we are facing is how can we conclude with a high degree of confidence that $\theta> 2$.

In this paper we propose the following point estimator of *θ* and will investigate its asymptotic properties:

$$\hat{\theta}_{n} = \frac{\log n}{\log\max_{1 \leq k \leq n} \vert X_{k} \vert }. $$

Here and below $\log x = \ln(e \vee x)$, $- \infty< x < \infty$.

Our main results will be stated in Sect. 2 and they all pertain to a sequence of i.i.d. random variables $\{X_{n}; n \geq1\}$ drawn from the distribution function $F(\cdot)$ of the random variable *X*. The proofs of our main results will be provided in Sect. 3.

## Statement of the main results

Throughout, *X* is a random variable with unknown distribution $F(x) = \mathbb{P}(X \leq x)$, $-\infty< x < \infty$ and write

$$\rho_{1} = \sup\Bigl\{ r \geq0: \lim_{x \rightarrow\infty} x^{r} \mathbb{P}(X > x) = 0 \Bigr\} \quad\mbox{and}\quad \rho_{2} = \sup\Bigl\{ r \geq0: \liminf_{x \rightarrow\infty} x^{r} \mathbb{P}(X > x) = 0 \Bigr\} . $$

Clearly, just as *θ* as defined in Sect. 1 is a parameter of the distribution $F(\cdot)$ of the random variable *X*, so are $\rho_{1}$ and $\rho_{2}$. These parameters satisfy

$$0 \leq\rho_{1} \leq\rho_{2} \leq\infty. $$

The main results of this paper are Theorems 2.1–2.5.

### Theorem 2.1

*Let* $\{X_{n}; n \geq1\}$ *be a sequence of i*.*i*.*d*. *random variables drawn from the distribution function* $F(\cdot)$ *of the random variable* *X*. *Then*

2.1 $$ \limsup_{n \rightarrow\infty} \frac{\log\max_{1 \leq k \leq n} X_{k}}{\log n} = \frac{1}{\rho_{1}}\quad\textit{a.s.} $$

*and there exists an increasing positive integer sequence* $\{l_{n}; n \geq1 \}$ (*which depends on the probability distribution of* *X* *when* $\rho_{1} < \infty$) *such that*

2.2 $$ \lim_{n \rightarrow\infty} \frac{\log\max_{1 \leq k \leq l_{n}} X_{k}}{\log l_{n}} = \frac{1}{\rho_{1}} \quad\textit{a.s.} $$

### Theorem 2.2

*Let* $\{X_{n}; n \geq1\}$ *be a sequence of i*.*i*.*d*. *random variables drawn from the distribution function* $F(\cdot)$ *of the random variable* *X*. *Then*

2.3 $$ \liminf_{n \rightarrow\infty} \frac{\log\max_{1 \leq k \leq n} X_{k}}{\log n} = \frac{1}{\rho_{2}}\quad\textit{a.s.} $$

*and there exists an increasing positive integer sequence* $\{m_{n}; n \geq1 \}$ (*which depends on the probability distribution of* *X* *when* $\rho_{2} > 0$) *such that*

2.4 $$ \lim_{n \rightarrow\infty} \frac{\log\max_{1 \leq k \leq m_{n}} X_{k}}{\log m_{n}} = \frac{1}{\rho_{2}} \quad\textit{a.s.} $$

### Remark 2.1

We must point out that (2.2) and (2.4) are two interesting conclusions. To see this, let $\{U_{n}; n \geq1 \}$ be a sequence of independent random variables with

$$\mathbb{P} (U_{n} = 1 ) = \mathbb{P} (U_{n} = 3 ) = \frac{1}{2n} \quad\mbox{and}\quad\mathbb{P} (U_{n} = 2 ) = 1 - \frac{1}{n}, \quad n \geq1. $$

Since

$$\sum_{n=1}^{\infty} \mathbb{P} (U_{n} = 3 ) = \sum_{n=1}^{\infty} \mathbb{P} (U_{n} = 1 ) = \sum_{n=1}^{\infty} \frac{1}{2n} = \infty, $$

it follows from the Borel–Cantelli lemma that

$$\limsup_{n \rightarrow\infty} U_{n} = 3\quad\mbox{a.s.}\quad \mbox{and}\quad\liminf_{n \rightarrow\infty} U_{n} = 1\quad \mbox{a.s.} $$

However, for any sequences $\{l_{n}; n \geq1\}$ and $\{m_{n}; n \geq1 \}$ of increasing positive integers,

$$\mbox{neither}\quad\lim_{n \rightarrow\infty} U_{l_{n}} = 3\quad \mbox{a.s.}\quad\mbox{nor}\quad\lim_{n \rightarrow\infty} U_{m_{n}} = 1 \quad\mbox{a.s.}\quad \mbox{holds}. $$

### Remark 2.2

For an observable random variable *X*, it is often the case that $\rho _{1} = \rho_{2}$. However, for any given constants $\rho_{1}$ and $\rho_{2}$ with $0 \leq\rho_{1} < \rho_{2} \leq\infty$, one can construct a random variable *X* such that

$$\sup\Bigl\{ r \geq0: \lim_{x \rightarrow\infty} x^{r} \mathbb{P}(X > x) = 0 \Bigr\} = \rho_{1} \quad\mbox{and}\quad\sup\Bigl\{ r \geq 0: \liminf_{x \rightarrow\infty} x^{r} \mathbb{P}(X > x) = 0 \Bigr\} = \rho_{2}. $$

For example, if $0 < \rho_{1} < \rho_{2} < \infty$, a random variable *X* can be constructed having probability distribution given by

$$\mathbb{P} (X = d_{n} ) = \frac{c}{d_{n}^{\rho_{1}}}, \quad n \geq1, $$

where $d_{n} = 2^{ (\rho_{2}/\rho_{1} )^{n}}$, $n \geq1$ and

$$c = \Biggl(\sum_{n = 1}^{\infty} \frac{1}{d_{n}^{\rho_{1}}} \Biggr)^{-1} > 0. $$

Combining Theorems 2.1 and 2.2, we establish a law of large numbers for $\log\max_{1 \leq k \leq n} X_{k}$, $n \geq1$ as follows.

### Theorem 2.3

*Let* $\{X_{n}; n \geq1\}$ *be a sequence of i*.*i*.*d*. *random variables drawn from the distribution function* $F(\cdot)$ *of the random variable* *X* *and let* $\rho\in[0, \infty]$. *Then the following four statements are equivalent*:

2.5 $$\begin{aligned}& \frac{\log\max_{1 \leq k \leq n} X_{k}}{\log n} \stackrel{\textit {a.s.}}{\longrightarrow} \frac{1}{\rho}, \end{aligned}$$

2.6 $$\begin{aligned}& \frac{\log\max_{1 \leq k \leq n} X_{k}}{\log n} \stackrel{\mathbb{P}}{\longrightarrow} \frac{1}{\rho}, \end{aligned}$$

2.7 $$\begin{aligned}& \rho_{1} = \rho_{2} = \rho, \end{aligned}$$

2.8 $$\begin{aligned}& \lim_{x \rightarrow\infty} x^{r} \mathbb{P}(X > x) = \textstyle\begin{cases} 0 & \forall r < \rho\textit{ if }\rho> 0,\\ \infty& \forall r > \rho\textit{ if } \rho< \infty. \end{cases}\displaystyle \end{aligned}$$

*If* $0 \leq\rho< \infty$, *then anyone of* (2.5)*–*(2.8) *holds if and only if there exists a function* $L(\cdot): (0, \infty) \rightarrow(0, \infty)$ *such that*

2.9 $$ \mathbb{P}(X > x) \sim\frac{L(x)}{x^{\rho}}\quad\textit {as } x \rightarrow\infty\quad\textit{and}\quad\lim_{x \rightarrow\infty} \frac{\ln L(x)}{\ln x} = 0. $$

The following result concerns convergence in distribution for $\log\max _{1 \leq k \leq n}X_{k}$, $n \geq1$.

### Theorem 2.4

*Let* $\{X_{n}; n \geq1\}$ *be a sequence of i*.*i*.*d*. *random variables drawn from the distribution function* $F(\cdot)$ *of the random variable* *X*. *Suppose that there exist constants* $0 < \rho< \infty$ *and* $-\infty< \tau< \infty$ *and a monotone function* $h(\cdot): [0, \infty) \rightarrow(0, \infty)$ *with* $\lim_{x \rightarrow\infty}h(x^{2})/h(x) = 1$ *such that*

2.10 $$ \mathbb{P}(X > x) \sim\frac{(\log x)^{\tau}h(x)}{x^{\rho }} \quad\textit{as } x \rightarrow\infty. $$

*Then*

2.11 $$ \begin{aligned}[b] &\lim_{n \rightarrow\infty} \mathbb{P} \biggl(\log\max _{1 \leq k \leq n} X_{k} \leq\frac{\ln n + \tau\ln\ln n + \ln h(n) - \tau\ln\rho+ x}{\rho} \biggr)\\ &\quad = \exp \bigl(-e^{-x} \bigr) \quad\forall- \infty< x < \infty. \end{aligned} $$

Also, by Theorems 2.1–2.3, we have the following result for the point estimator $\hat{\theta}_{n}$.

### Theorem 2.5

*Let* $\{X_{n}; n \geq1\}$ *be a sequence of i*.*i*.*d*. *random variables drawn from the distribution function* $F(\cdot)$ *of the random variable* *X*. *Let*

$$\hat{\theta}_{n} = \frac{\log n}{\log\max_{1 \leq k \leq n} \vert X_{k} \vert },\quad n \geq1. $$

*Then we have*

$$\begin{aligned}& \liminf_{n \rightarrow\infty} \hat{\theta}_{n} = \theta= \sup \bigl\{ r \geq0: \mathbb{E} \vert X \vert ^{r} < \infty\bigr\} \quad \textit{a.s.}, \\& \limsup_{n \rightarrow\infty} \hat{\theta}_{n} = \sup\Bigl\{ r \geq 0: \liminf_{x \rightarrow\infty} x^{r} \mathbb{P}\bigl( \vert X \vert > x\bigr) = 0 \Bigr\} \quad\textit{a.s.}, \end{aligned}$$

*and the following three statements are equivalent*:

2.12 $$\begin{aligned}& \hat{\theta}_{n} \stackrel{\textit{a.s.}}{\longrightarrow} \theta, \end{aligned}$$

2.13 $$\begin{aligned}& \hat{\theta}_{n} \stackrel{\mathbb{P}}{\longrightarrow} \theta, \end{aligned}$$

2.14 $$\begin{aligned}& \lim_{x \rightarrow\infty} x^{r} \mathbb{P}\bigl( \vert X \vert > x\bigr) = \infty\quad\forall r > \theta\textit{ if } \theta< \infty. \end{aligned}$$

*If* $0 \leq\theta< \infty$, *then anyone of* (2.12)*–*(2.14) *holds if and only if there exists a function* $L(\cdot): (0, \infty) \rightarrow(0, \infty)$ *such that*

2.15 $$ \mathbb{P}\bigl( \vert X \vert > x\bigr) \sim \frac{L(x)}{x^{\theta}}\quad\textit{as } x \rightarrow\infty\quad \textit{and}\quad\lim _{x \rightarrow\infty} \frac{\ln L(x)}{\ln x} = 0. $$

### Remark 2.3

Let $\{X_{n}; n \geq1\}$ be a sequence of i.i.d. random variables drawn from the distribution function $F(\cdot)$ of some nonnegative random variable *X*. For each $n \geq1$, let $X_{n,1} \leq X_{n,2} \leq\cdots\leq X_{n,n}$ denote the order statistics based on $X_{1}, X_{2}, \ldots, X_{n}$. To estimate the tail index of $F(\cdot)$, the well-known Hill estimator, proposed by Hill [1], is defined by

$$\hat{\alpha}_{n} = \Biggl(\frac{1}{k_{n}} \sum _{i=1}^{k_{n}} \ln\frac{X_{n,n-i+1}}{X_{n,n-k_{n}}} \Biggr)^{-1}, $$

where $\{k_{n}; n \geq1 \}$ is a sequence of positive integers satisfying

2.16 $$ 1 \leq k_{n} < n\quad\mbox{and}\quad k_{n} \rightarrow\infty\quad\mbox{and}\quad k_{n}/n \rightarrow0\quad \mbox{as } n \rightarrow\infty. $$

Mason [2, Theorem 2] showed that, for some constant $\theta\in (0, \infty)$,

$$\hat{\alpha}_{n} \stackrel{\mathbb{P}}{\longrightarrow} \theta\quad \mbox{for every sequence } \{k_{n}; n \geq1 \} \mbox{ satisfying (2.16)} $$

if and only if

2.17 $$ \begin{aligned}[b] &\mathbb{P}(X > x) \sim\frac{L(x)}{x^{\theta}}\\ &\quad\mbox{as } x \rightarrow\infty\mbox{ where } L(\cdot): (0, \infty) \rightarrow(0, \infty) \mbox{ is a slowly varying function}. \end{aligned} $$

Since $L(\cdot)$ defined in (2.17) is a slowly varying function,

$$\lim_{t \rightarrow\infty} \frac{\log L(t)}{\log t} = 0 $$

is always true and hence (2.15) follows from (2.17). However, the following example shows that (2.15) does not imply (2.17). Thus condtion (2.15) is weaker than (2.17).

### Example 2.1

Let $\{X_{n}; n \geq1\}$ be a sequence of i.i.d. random variables drawn from the distribution function $F(\cdot)$ of some nonnegative random variable *X* given by

$$F(x) = 1 - \exp\bigl(- \theta\bigl[\ln(x \vee1)\bigr] \bigr), \quad x \geq 0, $$

where $\theta\in(0, \infty)$ is the tail index of the distribution and $[t]$ denotes the integer part of *t*. Then (2.15) holds but (2.17) is not satisfied. To see this, let

$$L(x) = \exp\bigl(\theta\bigl(\ln x - [\ln x] \bigr) \bigr), \quad x \geq e. $$

Then

$$\mathbb{P}(X > x) = 1 - F(x) = x^{-\theta} L(x), \quad x \geq e. $$

Since, for $x \geq e$, $0 \leq\ln x -[\ln x] \leq1$, we have

$$1 \leq L(x) \leq\exp(\theta), \quad x \geq1 $$

and hence (2.15) holds. However, for $1 < a < e$ and $x_{n} = e^{n}$, $n \geq1$, we have

$$\ln(ax_{n} ) - \bigl[\ln(a x_{n} ) \bigr] = (n + \ln a ) - [ n + \ln a ] = \ln a\quad\mbox{and}\quad\ln(x_{n} ) - \bigl[\ln (x_{n} ) \bigr] = n - [ n ] = 0. $$

Thus, for $\theta\in(0, \infty)$,

$$\frac{L (ax_{n} )}{L (x_{n} )} = \frac{\exp(\theta(\ln a))}{\exp(\theta \times0)} = a^{\theta} > 1, \quad n \geq1 $$

and hence

$$\lim_{x \rightarrow\infty} \frac{L(ax)}{L(x)} = 1\quad\mbox{does not hold}; $$

i.e., $L(\cdot)$ is not a slowly varying function. Thus (2.17) is not satisfied and hence, for this example, the well-known Hill estimator cannot be used to estimate the tail index *θ*.

## Proofs of the main results

Let $\{A_{n}; n \geq1 \}$ be a sequence of events. As usual the abbreviation $\{A_{n} \mbox{ i.o.} \}$ stands for the case that the events $A_{n}$ occur infinitely often. That is,

$$\{A_{n} \mbox{ i.o.} \} = \{\mbox{events } A_{n} \mbox{ occur infinitely often} \} = \bigcap_{n=1}^{\infty} \bigcup_{j=n}^{\infty} A_{j}. $$

For events *A* and *B*, we say $A = B$ a.s. if $\mathbb{P}(A \mathrel{\Delta} B) = 0$ where $A \mathrel{\Delta}B = (A \setminus B) \cup(B \setminus A)$. To prove Theorem 2.1, we use the following preliminary result, which can be found in Chandra [3, Example 1.6.25(a), p. 48].

### Lemma 3.1

*Let* $\{b_{n}; n \geq1 \}$ *be a nondecreasing sequence of positive real numbers such that*

$$\lim_{n \rightarrow\infty} b_{n} = \infty $$

*and let* $\{V_{n}; n \geq1 \}$ *be a sequence of random variables*. *Then*

$$\Bigl\{ \max_{1 \leq k \leq n} V_{k} \geq b_{n} \textit{ i.o.} \Bigr\} = \{ V_{n} \geq b_{n} \textit{ i.o.} \} \quad\textit{a.s.} $$

### Proof of Theorem 2.1

*Case I:* $0 < \rho_{1} < \infty$*.* For given $\epsilon> 0$, let $r(\epsilon) = (\frac{1}{\rho_{1}} + \epsilon )^{-1}$. Then

$$0 < r(\epsilon) < \rho_{1} = \sup\Bigl\{ r \geq0: \lim _{x \rightarrow\infty} x^{r} \mathbb{P}(X > x) = 0 \Bigr\} $$

and hence

3.1 $$ \sum_{n = 1}^{\infty} \mathbb{P} \bigl(X > n^{1/r(\epsilon)} \bigr) < \infty. $$

By the Borel–Cantelli lemma, (3.1) implies that

$$\mathbb{P} \bigl(X_{n} > n^{1/r(\epsilon)} \mbox{ i.o.} \bigr) = 0. $$

By Lemma 3.1, we have

$$\biggl\{ \frac{\log\max_{1 \leq k \leq n}X_{k}}{\log n} > \frac{1}{\rho _{1}} + \epsilon\mbox{ i.o.} \biggr\} = \Bigl\{ \max_{1 \leq k \leq n} X_{k} > n^{1/r(\epsilon)} \mbox{ i.o.} \Bigr\} = \bigl\{ X_{n} > n^{1/r(\epsilon)} \mbox{ i.o.} \bigr\} \quad\mbox{a.s.} $$

and hence

$$\mathbb{P} \biggl( \frac{\log\max_{1 \leq k \leq n}X_{k}}{\log n} > \frac{1}{\rho_{1}} + \epsilon\mbox{ i.o.} \biggr) = 0. $$

Thus

$$\limsup_{n \rightarrow\infty} \frac{\log\max_{1 \leq k \leq n}X_{k}}{\log n} \leq\frac{1}{\rho_{1}} + \epsilon\quad\mbox{a.s.} $$

Letting $\epsilon\searrow0$, we get

3.2 $$ \limsup_{n \rightarrow\infty} \frac{\log\max_{1 \leq k \leq n}X_{k}}{\log n} \leq \frac{1}{\rho_{1}}\quad\mbox{a.s.} $$

By the definition of $\rho_{1}$, we have

$$\limsup_{x \rightarrow\infty} x^{r} \mathbb{P}(X > x) = \infty\quad \forall r > \rho_{1}, $$

which is equivalent to

$$\limsup_{x \rightarrow\infty} x \mathbb{P} \bigl(X > x^{(1/\rho_{1}) - \epsilon} \bigr) = \infty\quad\forall\epsilon> 0. $$

Then, inductively, we can choose positive integers $l_{n}$, $n \geq1$ such that

$$1 = l_{1} < l_{2} < \cdots< l_{n} < \cdots \quad\mbox{and}\quad l_{n}\mathbb{P} \bigl(X > l_{n}^{(1/\rho_{1}) - (1/n)} \bigr) \geq2 \ln n, \quad n \geq1. $$

Note that, for any $0 \leq z \leq1$, $1 - z \leq e^{-z}$. Thus, for all sufficiently large *n*, we have

$$\begin{aligned} \mathbb{P} \biggl(\frac{\log\max_{1 \leq k \leq l_{n}} X_{k}}{\log l_{n}} \leq\frac{1}{\rho_{1}} - \frac{1}{n} \biggr) &= \mathbb{P} \Bigl(\max_{1 \leq k \leq l_{n}}X_{k} \leq l_{n}^{(1/\rho_{1}) - (1/n)} \Bigr) \\ &= \bigl(1 - \mathbb{P} \bigl(X > l_{n}^{(1/\rho_{1}) - (1/n)} \bigr) \bigr)^{l_{n}} \\ &\leq\exp\bigl(- l_{n}\mathbb{P} \bigl(X > l_{n}^{(1/\rho_{1}) - (1/n)} \bigr) \bigr) \\ &\leq\exp(-2 \ln n) \\ &= n^{-2}. \end{aligned} $$

Since $\sum_{n=1}^{\infty} n^{-2} < \infty$, by the Borel–Cantelli lemma, we get

$$\mathbb{P} \biggl(\frac{\log\max_{1 \leq k \leq l_{n}} X_{k}}{\log l_{n}} \leq\frac{1}{\rho_{1}} - \frac{1}{n} \mbox{ i.o.} \biggr) = 0 $$

which ensures that

3.3 $$ \liminf_{n \rightarrow\infty} \frac{\log\max_{1 \leq k \leq l_{n}} X_{k}}{\log l_{n}} \geq \frac{1}{\rho_{1}}\quad\mbox{a.s.} $$

Clearly, (2.1) and (2.2) follow from (3.2) and (3.3).

*Case II:* $\rho_{1} = \infty$*.* Using the same argument used in the first half of the proof for Case I, we get

$$\limsup_{n \rightarrow\infty} \frac{\log\max_{1 \leq k \leq n} X_{k}}{\log n} \leq\epsilon\quad\mbox{a.s. } \forall\epsilon> 0 $$

and hence

3.4 $$ \limsup_{n \rightarrow\infty} \frac{\log\max_{1 \leq k \leq n} X_{k}}{\log n} \leq0\quad \mbox{a.s.} $$

Note that

$$0 \leq\frac{\log\max_{1 \leq k \leq n} X_{k}}{\log n}\quad\forall n \geq1. $$

We thus have

3.5 $$ \liminf_{n \rightarrow\infty} \frac{\log\max_{1 \leq k \leq n} X_{k}}{\log n} \geq0 \quad \mbox{a.s.} $$

It thus follows from (3.4) and (3.5) that

$$\lim_{n \rightarrow\infty} \frac{\log\max_{1 \leq k \leq n} X_{k}}{\log n} = 0 \quad\mbox{a.s.} $$

proving (2.1) and (2.2) (with $l_{n} = n$, $n \geq1$).

*Case III:* $\rho_{1} = 0$*.* By the definition of $\rho_{1}$, we have

$$\limsup_{x \rightarrow\infty} x^{r} \mathbb{P}(X > x) = \infty\quad \forall r > 0, $$

which is equivalent to

$$\limsup_{x \rightarrow\infty} x \mathbb{P} \bigl(X > x^{r} \bigr) = \infty\quad\forall r > 0. $$

Then, inductively, we can choose positive integers $l_{n}$, $n \geq1$ such that

$$1 = l_{1} < l_{2} < \cdots< l_{n} < \cdots \quad\mbox{and}\quad l_{n}\mathbb{P} \bigl(X > l_{n}^{n} \bigr) \geq2 \ln n, \quad n \geq1. $$

Thus, for all sufficiently large *n*, we have by the same argument as in Case I

$$\mathbb{P} \biggl(\frac{\log\max_{1 \leq k \leq l_{n}}X_{k}}{\log l_{n}} \leq n \biggr) \leq n^{-2} $$

and hence by the Borel–Cantelli lemma

$$\mathbb{P} \biggl(\frac{\log\max_{1 \leq k \leq l_{n}} X_{k}}{\log l_{n}} \leq n \mbox{ i.o.} \biggr) = 0 $$

which ensures that

$$\lim_{n \rightarrow\infty} \frac{\log\max_{1 \leq k \leq l_{n}} X_{k}}{\log l_{n}} = \infty\quad\mbox{a.s.} $$

Thus (2.1) and (2.2) hold. This completes the proof of Theorem 2.1. □

### Proof of Theorem 2.2

*Case I:* $0 < \rho_{2} < \infty$*.* For given $\rho_{2} < r < \infty$, let $r_{1} = (r + \rho_{2} )/2$ and $\tau= 1 - (r_{1}/r)$. Then $\rho _{2} < r_{1} < r < \infty$ and $\tau> 0$. By the definition of $\rho_{2}$, we have

$$\lim_{x \rightarrow\infty} x^{r_{1}} \mathbb{P}(X > x) = \infty $$

and hence, for all sufficiently large *x*,

$$\mathbb{P}(X > x) \geq x^{-r_{1}}. $$

Thus, for all sufficiently large *n*,

$$n \mathbb{P} \bigl(X > n^{1/r} \bigr) \geq n \bigl(n^{1/r} \bigr)^{-r_{1}} = n^{1 - (r_{1}/r)} = n^{\tau} $$

and hence

$$\mathbb{P} \Bigl(\max_{1 \leq k \leq n}X_{k} \leq n^{1/r} \Bigr) = \bigl(1 - \mathbb{P} \bigl(X > n^{1/r} \bigr) \bigr)^{n} \leq e^{-n \mathbb{P} (X > n^{1/r} )} \leq e^{-n^{\tau}}. $$

Since

$$\sum_{n=1}^{\infty} e^{-n^{\tau}} < \infty, $$

by the Borel–Cantelli lemma, we have

$$\mathbb{P} \Bigl(\max_{1 \leq k \leq n} X_{k} \leq n^{1/r} \mbox{ i.o.} \Bigr) = 0, $$

which implies that

$$\liminf_{n \rightarrow\infty} \frac{\log\max_{1 \leq k \leq n}X_{k}}{\log n} \geq1/r \quad\mbox{a.s.} $$

Letting $r \searrow\rho_{2}$, we get

3.6 $$ \liminf_{n \rightarrow\infty} \frac{\log\max_{1 \leq k \leq n}X_{k}}{\log n} \geq \frac{1}{\rho_{2}}\quad\mbox{a.s.} $$

Again, by the definition of $\rho_{2}$, we have

$$\liminf_{x \rightarrow\infty} x^{r} \mathbb{P}(X > x) = 0\quad \forall r < \rho_{2}, $$

which is equivalent to

$$\liminf_{x \rightarrow\infty} x \mathbb{P} \bigl(X > x^{(1/\rho_{2}) + \epsilon} \bigr) = 0\quad\forall\epsilon> 0. $$

Then, inductively, we can choose positive integers $m_{n}$, $n \geq1$ such that

$$1 = m_{1} < m_{2} < \cdots< m_{n} < \cdots \quad\mbox{and}\quad m_{n}\mathbb{P} \bigl(X > m_{n}^{(1/\rho_{2}) + (1/n)} \bigr) \leq n^{-2}, \quad n \geq1. $$

Then we have

$$\sum_{n=1}^{\infty} \mathbb{P} \Bigl(\max _{1 \leq k \leq m_{n}}X_{k} > m_{n}^{(1/\rho_{2}) + (1/n)} \Bigr) \leq\sum_{n=1}^{\infty} m_{n} \mathbb{P} \bigl(X > m_{n}^{(1/\rho_{2}) + (1/n)} \bigr) \leq\sum _{n=1}^{\infty} n^{-2} < \infty. $$

Thus, by the Borel–Cantelli lemma, we get

$$\mathbb{P} \biggl(\frac{\log\max_{1 \leq k \leq m_{n}} X_{k}}{\log m_{n}} > \frac{1}{\rho_{2}} + \frac{1}{n} \mbox{ i.o.} \biggr) = 0 $$

which ensures that

3.7 $$ \limsup_{n \rightarrow\infty} \frac{\log\max_{1 \leq k \leq m_{n}} X_{k}}{\log m_{n}} \leq \frac{1}{\rho_{2}}\quad\mbox{a.s.} $$

Clearly, (2.3) and (2.4) follow from (3.6) and (3.7).

*Case II:* $\rho_{2} = \infty$*.* By the definition of $\rho_{2}$, we have

$$\liminf_{x \rightarrow\infty} x^{r} \mathbb{P}(X > x) = 0\quad \forall r > 0, $$

which is equivalent to

$$\liminf_{x \rightarrow\infty} x \mathbb{P} \bigl(X > x^{r} \bigr) = 0\quad\forall r > 0. $$

Then, inductively, we can choose positive integers $m_{n}$, $n \geq1$ such that

$$1 = m_{1} < m_{2} < \cdots< m_{n} < \cdots \quad\mbox{and}\quad m_{n}\mathbb{P} \bigl(X > m_{n}^{1/n} \bigr) \leq n^{-2}, \quad n \geq1. $$

Thus

$$\sum_{n=1}^{\infty} \mathbb{P} \Bigl(\max _{1 \leq k \leq m_{n}}X_{k} > m_{n}^{1/n} \Bigr) \leq\sum_{n=1}^{\infty} m_{n} \mathbb{P} \bigl(X > m_{n}^{1/n} \bigr) \leq\sum _{n=1}^{\infty} n^{-2} < \infty $$

and hence by the Borel–Cantelli lemma

$$\mathbb{P} \Bigl( \max_{1 \leq k \leq m_{n}} X_{k} > m_{n}^{1/n} \mbox{ i.o.} \Bigr) = 0, $$

which ensures that

3.8 $$ \limsup_{n \rightarrow\infty} \frac{\log\max_{1 \leq k \leq m_{n}} X_{k}}{\log m_{n}} \leq0\quad \mbox{a.s.} $$

It is clear that

3.9 $$ \liminf_{n \rightarrow\infty} \frac{\log\max_{1 \leq k \leq n} X_{k}}{\log n} \geq0\quad \mbox{a.s.} $$

It thus follows from (3.8) and (3.9) that

$$\liminf_{n \rightarrow\infty} \frac{\log\max_{1 \leq k \leq n} X_{k}}{\log n} = 0\quad\mbox{a.s.} \quad \mbox{and}\quad\lim_{n \rightarrow\infty} \frac{\log\max_{1 \leq k \leq m_{n}} X_{k}}{\log m_{n}} = 0\quad \mbox{a.s.;} $$

i.e., (2.3) and (2.4) hold.

*Case III:* $\rho_{2} = 0$*.* Using the same argument used in the first half of the proof for Case I, we get

$$\liminf_{n \rightarrow\infty} \frac{\log\max_{1 \leq k \leq n} X_{k}}{\log n} \geq\frac{1}{r}\quad \mbox{a.s. } \forall r > 0. $$

Letting $r \searrow0$, we get

$$\liminf_{n \rightarrow\infty} \frac{\log\max_{1 \leq k \leq n} X_{k}}{\log n} = \infty\quad\mbox{a.s.} $$

Thus

$$\lim_{n \rightarrow\infty} \frac{\log\max_{1 \leq k \leq n} X_{k}}{\log n} = \infty\quad\mbox{a.s.} $$

and hence (2.3) and (2.4) hold with $m_{n} = n$, $n \geq1$. □

### Proof of Theorem 2.3

It follows from Theorems 2.1 and 2.2 that

$$(2.5) \Longleftrightarrow(2.7) \Longleftrightarrow (2.8). $$

Since (2.6) follows from (2.5), we only need to show that (2.6) implies (2.8). It follows from (2.6) that

3.10 $$ \lim_{n \rightarrow\infty} \mathbb{P} \biggl( \frac {\log\max_{1 \leq k \leq n} X_{k}}{\log n} \leq\frac{1}{r} \biggr) = \textstyle\begin{cases} 1 & \forall r < \rho\mbox{ if } \rho> 0,\\ 0 & \forall r > \rho\mbox{ if } \rho< \infty. \end{cases} $$

Since, for $n \geq3$,

$$\mathbb{P} \biggl( \frac{\log\max_{1 \leq k \leq n} X_{k}}{\log n} \leq\frac{1}{r} \biggr) = \mathbb{P} \Bigl( \max_{1 \leq k \leq n} X_{k} \leq n^{1/r} \Bigr) = \bigl(1 - \mathbb{P} \bigl(X > n^{1/r} \bigr) \bigr)^{n} = e^{n \ln (1 - \mathbb{P} (X > n^{1/r} ) )} $$

and

$$n \ln\bigl(1 - \mathbb{P} \bigl(X > n^{1/r} \bigr) \bigr) \sim- n \mathbb{P} \bigl(X > n^{1/r} \bigr)\quad\mbox{as } n \rightarrow\infty, $$

it follows from (3.10) that

$$\lim_{n \rightarrow\infty} n \mathbb{P} \bigl(X > n^{1/r} \bigr) = \textstyle\begin{cases} 0 & \forall r < \rho\mbox{ if } \rho> 0,\\ \infty& \forall r > \rho\mbox{ if } \rho< \infty, \end{cases} $$

which is equivalent to (2.8).

For $0 \leq\rho< \infty$, note that

$$\mathbb{P} (X > x) = x^{-\rho} \bigl(x^{\rho}\mathbb{P}(X > x) \bigr) = e^{-\rho\ln x + \ln (x^{\rho} \mathbb{P}(X > x) )}\quad \forall x > 0. $$

We thus see that, if $0 \leq\rho< \infty$, then (2.8) is equivalent to

$$\lim_{x \rightarrow\infty} \frac{\ln (x^{\rho} \mathbb{P}(X > x) )}{\log x} = 0. $$

(We leave it to the reader to work out the details of the proof.) We thus see that (2.8) implies (2.9) with $L(x) = x^{\rho} \mathbb{P}(X > x)$, $x > 0$. It is easy to verify that (2.8) follows from (2.9). This completes the proof of Theorem 2.3. □

### Proof of Theorem 2.4

For fixed $x \in(-\infty, \infty)$, write

$$a_{n}(x) = \frac{\ln n + \tau\ln\ln n + \ln h(n) - \tau\ln\rho+ x}{\rho}\quad\mbox{and}\quad b_{n}(x) = e^{a_{n}(x)}, n \geq2. $$

Then

$$b_{n}(x) = n^{1/\rho} (\ln n)^{\tau/\rho}\bigl(h(n) \bigr)^{1/\rho} \rho^{-\tau/\rho} e^{x/\rho}, n \geq2. $$

Since $h(\cdot): [0, \infty) \rightarrow(0, \infty)$ is a monotone function with $\lim_{x \rightarrow\infty}h(x^{2})/h(x) = 1$, $h(\cdot)$ is a slowly varying function such that $\lim_{x \rightarrow \infty} h(x^{r})/h(x) = 1$ $\forall r > 0$ and hence

$$h \bigl(b_{n}(x) \bigr) \sim h(n) \quad\mbox{as } n \rightarrow \infty. $$

Clearly,

$$\bigl(\ln b_{n}(x) \bigr)^{\tau} \sim\rho^{-\tau}(\ln n)^{\tau}\quad\mbox{as } n \rightarrow\infty. $$

It thus follows from (2.10) that, as $n \rightarrow\infty$,

$$\begin{aligned} n \ln\bigl(1 - \mathbb{P} \bigl(X > b_{n}(x) \bigr) \bigr) & \sim-n \mathbb{P} \bigl(X > b_{n}(x) \bigr) \\ & \sim-n \times\frac{ (\ln(b_{n}(x) ) )^{\tau} h (b_{n}(x) )}{ (b_{n}(x) )^{\rho}} \\ & \sim-n \times\frac{\rho^{-\tau}(\ln n)^{\tau} h(n)}{n (\ln n)^{\tau } h(n) \rho^{-\tau} e^{x}} \\ &= -e^{-x} \end{aligned} $$

so that

$$\begin{aligned} \lim_{n \rightarrow\infty} \mathbb{P} \Bigl(\log\max _{1 \leq k \leq n} X_{k} \leq a_{n}(x) \Bigr) &=\lim _{n \rightarrow\infty} \mathbb{P} \Bigl(\max_{1 \leq k \leq n} X_{k} \leq b_{n}(x) \Bigr) \\ &=\lim_{n \rightarrow\infty} \bigl(1 - \mathbb{P} \bigl(X > b_{n}(x) \bigr) \bigr)^{n} \\ &=\lim_{n \rightarrow\infty} e^{n \ln (1 - \mathbb{P} (X > b_{n}(x) ) )} \\ &=\exp\bigl(-e^{-x} \bigr); \end{aligned} $$

i.e., (2.11) holds. □

### Proof of Theorem 2.5

Since $\hat{\theta}_{n} = \frac{\log n}{\log\max_{1 \leq k \leq n} \vert X_{k} \vert }$, $n \geq1$, Theorem 2.5 follows immediately from Theorems 2.1–2.3. □

## Conclusions

In this paper we propose the following simple point estimator of *θ*, the power of moments of the random variable *X*, and investigate its asymptotic properties:

$$\hat{\theta}_{n} = \frac{\log n}{\log\max_{1 \leq k \leq n} \vert X_{k} \vert }. $$

In particular, we show that

$$\hat{\theta}_{n} \rightarrow_{\mathbb{P}} \theta\quad\mbox{if and only if}\quad\lim_{x \rightarrow\infty} x^{r} \mathbb{P}\bigl( \vert X \vert > x\bigr) = \infty\quad\forall r > \theta. $$

This means that, under very reasonable conditions on $F(\cdot)$, $\hat {\theta}_{n}$ is actually a consistent estimator of *θ*. From Remark 2.3 and Example 2.1, we see that, for a nonnegative random variable *X*, $\hat{\theta}_{n}$ is a consistent estimator of *θ* whenever the well-known Hill estimator $\hat{\alpha}_{n}$ is a consistent estimator of *θ*. However, the converse is not true.
